# Potential of ileal bile acid transporter inhibition as a therapeutic target in Alagille syndrome and progressive familial intrahepatic cholestasis

**DOI:** 10.1111/liv.14553

**Published:** 2020-06-22

**Authors:** Binita M. Kamath, Philip Stein, Roderick H. J. Houwen, Henkjan J. Verkade

**Affiliations:** ^1^ The Hospital for Sick Children Toronto ON Canada; ^2^ University of Toronto Toronto ON Canada; ^3^ Albireo Pharma, Inc Boston MA USA; ^4^ Wilhelmina Children’s Hospital University Medical Center Utrecht Netherlands; ^5^ University of Groningen Beatrix Children’s Hospital/University Medical Center Groningen Groningen The Netherlands

**Keywords:** bile acids and salts, cholestasis, paediatrics, pruritus, sodium‐bile acid cotransporter

## Abstract

Alagille syndrome (ALGS) and progressive familial intrahepatic cholestasis (PFIC) are rare, inherited cholestatic liver disorders that manifest in infants and children and are associated with impaired bile flow (ie cholestasis), pruritus and potentially fatal liver disease. There are no effective or approved pharmacologic treatments for these diseases (standard medical treatments are supportive only), and new, noninvasive options would be valuable. Typically, bile acids undergo biliary secretion and intestinal reabsorption (ie enterohepatic circulation). However, in these diseases, disrupted secretion of bile acids leads to their accumulation in the liver, which is thought to underlie pruritus and liver‐damaging inflammation. One approach to reducing pathologic bile acid accumulation in the body is surgical biliary diversion, which interrupts the enterohepatic circulation (eg by diverting bile acids to an external stoma). These procedures can normalize serum bile acids, reduce pruritus and liver injury and improve quality of life. A novel, nonsurgical approach to interrupting the enterohepatic circulation is inhibition of the ileal bile acid transporter (IBAT), a key molecule in the enterohepatic circulation that reabsorbs bile acids from the intestine. IBAT inhibition has been shown to reduce serum bile acids and pruritus in trials of paediatric cholestatic liver diseases. This review explores the rationale of inhibition of the IBAT as a therapeutic target, describes IBAT inhibitors in development and summarizes the current data on interrupting the enterohepatic circulation as treatment for cholestatic liver diseases including ALGS and PFIC.

**Abbreviations**
ABCB11: ATP‐binding cassette, sub‐family B, member 11 protein
ALGS: Alagille syndrome
ATP8B1: ATPase phospholipid transporting 8B1 protein
BSEP: bile salt export pump
C4: 7α‐hydroxy‐4‐cholesten‐3‐one
ChiLDReN: *Chi*ldhood *L*iver *D*isease *Re*search *N*etwork
FGF19: fibroblast growth factor
FXR: farnesoid X receptor
GALA: *G*lobal *AL*agille *A*lliance
IBAT: ileal bile acid transporter
*JAGGED1*: gene encoding a ligand of the Notch signalling pathway
NAPPED: *NA*tural course and *P*rognosis of *P*FIC and *E*ffect of biliary *D*iversion
NASH: nonalcoholic steatohepatitis
norUDCA: norursodeoxycholic acid
PBC: primary biliary cholangitis
PEBD: partial external biliary diversion
PFIC: progressive familial intrahepatic cholestasis
PSC: primary sclerosing cholangitis
SBD: surgical biliary diversion
UDCA: ursodeoxycholic acid

**Key points**

- Patients with the cholestatic liver diseases Alagille syndrome (ALGS) and progressive familial intrahepatic cholestasis (PFIC) have high disease burden and unmet medical needs.
- Currently, the only truly effective treatments for ALGS and PFIC are surgeries that disrupt the enterohepatic circulation of bile acids and liver transplantation.
- A number of potential treatment alternatives are in development to target mechanisms of cholestatic liver disease noninvasively, including ileal bile acid transport (IBAT) inhibitors.
- Phase 2 and 3 trial data suggest that IBAT inhibitors, which act by interrupting the enterohepatic circulation, may be safe and efficacious treatment options for ALGS and PFIC.

## 1 INTRODUCTION

**Figure 1:**
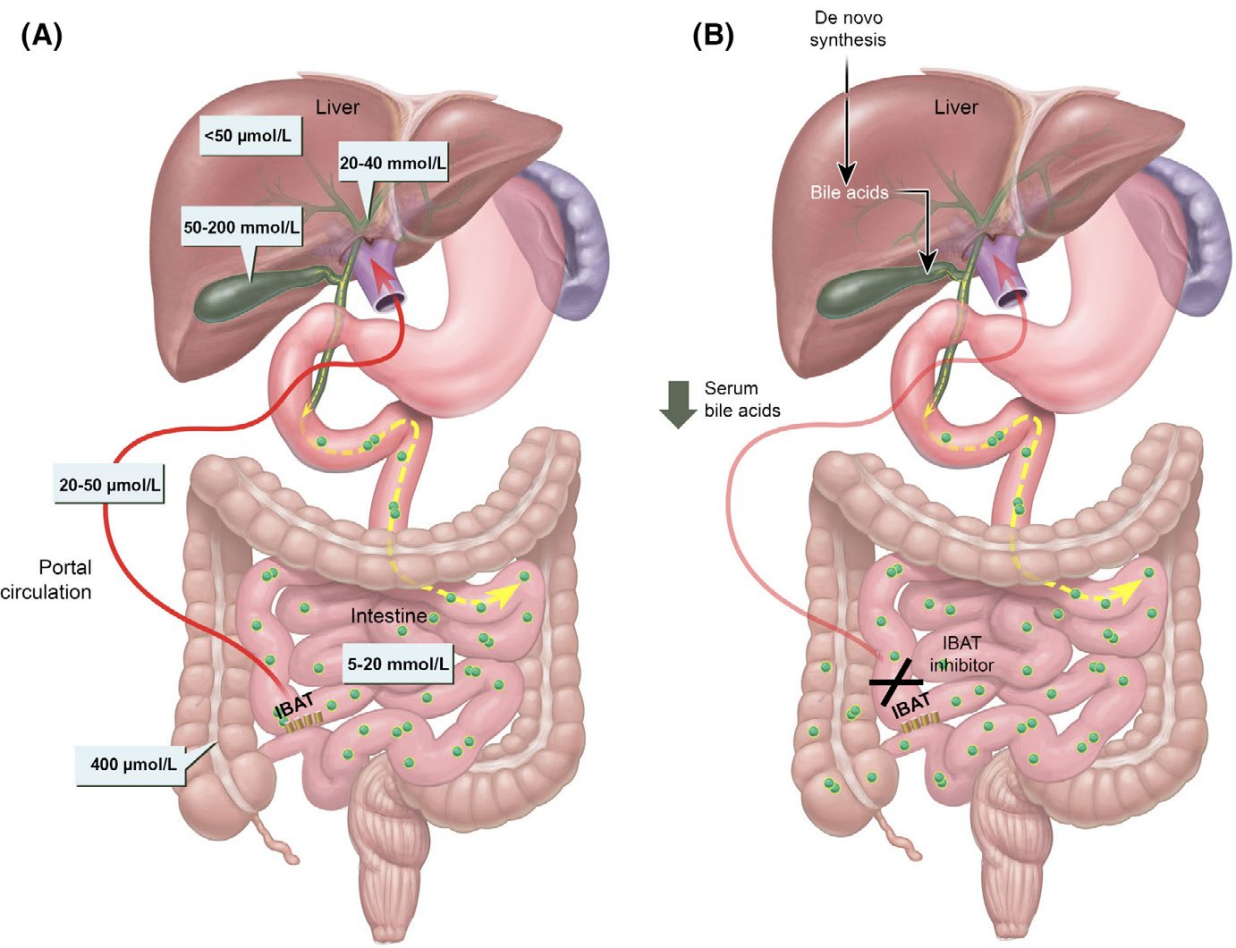
Role of IBAT, bile acids and enterohepatic circulation in homeostasis and disease. A, Bile acids, synthesized in and secreted from the liver, travel to the small intestine where they aid in digestion and absorption of nutrients. Bile acids are reabsorbed from the terminal ileum by IBAT (95%) and return to the liver through the portal veins (indicated by the red line). This cycle is known as enterohepatic circulation. Bile acids not recovered in this process are replaced by nascent synthesis (5%), which is governed by inhibitory feedback from FGF19. The synthesis intermediate C4 is frequently used as a readout of bile acid synthesis. High bile acid levels in the ileum prompt FGF19 signalling, which suppresses further bile acid production (indicated by a decrease in C4 levels). Typical bile acid concentrations in liver cells, the biliary and intestinal tracts and the portal circulation are given in milli‐ or micromolar quantities, as applicable.^1^ B, Pharmacologic inhibition of IBAT (the ileal bile acid transporter), a novel strategy being explored as treatment for Alagille syndrome and progressive familial intrahepatic cholestasis, prevents the recirculation of bile acids, shunting them away from the liver and towards faecal excretion instead, which is expected to reduce the overall size of the bile acid pool. C4 (7α‐hydroxy‐4‐cholesten‐3‐one), bile acid precursor; FGF19, fibroblast growth factor 19^3^

Adequate enterohepatic circulation is crucial for homeostatic maintenance of the bile acid pool in the body. This process starts with bile acid synthesis in hepatocytes and their subsequent biliary secretion, primarily mediated by the bile salt export pump (BSEP) at the apical (canalicular) membrane. Bile salts then move through bile ducts as constituents of bile to the gallbladder for storage and, later, for release into the small intestine to aid in lipid digestion and absorption. Per cycle, up to 95% of bile acids are reabsorbed from the terminal ileal lumen by the ileal bile acid transporter (IBAT; also known as the apical sodium‐dependent bile acid transporter) for return to the liver through the portal veins (Figure 1A).^1^ Bile acid export from hepatocytes may occur at the basolateral membrane, which directs bile acids into systemic circulation. Under physiologic conditions, this export pathway is minimal; however, this may be enhanced in certain situations as a hepatoprotective mechanism, such as during cholestasis.^2^, ^3^ Bile acids that are not recovered from the intestine are lost in faeces (~5%) and, under steady‐state conditions, are replaced by hepatic de novo synthesis.

The bile acid pool size is regulated by feedback loops: the nuclear sensor farnesoid X receptor (FXR) responds to bile acid concentrations at various points along the enterohepatic circulation by signalling to repress the synthesis of additional bile acids. For example, after bile acid reuptake from the intestinal lumen at the level of the terminal ileum, the bile acids activate FXR and thereby FXR‐responsive genes like fibroblast growth factor 19 (FGF19); once expressed, FGF19 protein is secreted and relays information from enterocytes to the liver, such that enzymes involved in the synthesis of bile acids (eg cholesterol 7 alpha‐hydroxylase) are subsequently repressed. Bile acid synthesis is commonly quantified by the serum levels of the intermediate synthesis product C4 (7α‐hydroxy‐4‐cholesten‐3‐one), which tends to negatively correlate with FGF19 levels (ie bile acid accumulation drives FGF19 formation, resulting in lower C4 levels [and bile acid synthesis; Figure  1A]^1^; the converse is also true: diminished intestinal FXR activation by bile acids decreases FGF19 production, leading to enhanced bile acid synthesis and higher C4 levels).^4^, ^5^

Cholestasis is defined as the impaired formation or flow of bile in the hepatobiliary system and may be intrahepatic (involving hepatocytes, bile canaliculi or intrahepatic bile ducts) or extrahepatic (involving the bile ducts outside the liver or the gallbladder).^6^, ^7^, ^8^ In cholestasis, which can present with features of jaundice or pruritus, the accumulation of bile acids may damage liver cells such that fibrotic and inflammatory pathways are activated that lead to liver injury.^1^, ^2^, ^6^ Common cholestatic liver diseases include Alagille syndrome (ALGS), progressive familial intrahepatic cholestasis (PFIC) and biliary atresia in children and primary biliary cholangitis (PBC) or primary sclerosing cholangitis (PSC) in adults.

This review focuses on ALGS and PFIC, which are inherited and severe intrahepatic cholestatic liver diseases in children. This review will explore the therapeutic potential of interrupting the enterohepatic circulation via pharmacologic blockade of IBAT and the associated implications for treating cholestatic liver diseases and other disorders.

## 2 OVERVIEW OF ALGS AND PFIC

ALGS and PFIC are genetic diseases that can present in paediatric patients as severe cholestasis and result from intrahepatic perturbations.^6^, ^7^ ALGS can be characterized by a reduction of intrahepatic bile ducts (in association with abnormalities in a number of non‐liver organ systems such as heart defects, dysmorphic facial features, and vascular, vertebral and ocular anomalies),^9^, ^10^ with clinical severity that ranges from biochemical liver abnormalities only to end‐stage liver disease.^10^, ^11^ PFIC represents a group of disorders (with subtypes grouped based on the underlying genetic deficiency; eg ATP8B1‐deficient PFIC, ABCB11‐deficient PFIC) in which disruption of bile homeostasis can eventually lead to cholestasis, cirrhosis, liver failure and death.^12^, ^13^, ^14^ Although distinctly different in many aspects, patients with ALGS and PFIC can share common clinical traits such as cholestasis, pruritus and an eventual need for liver transplantation.^15^, ^16^ Table 1 provides additional details on the incidence, genetic basis, proposed mechanisms of disease, clinical presentation and disease progression of ALGS and PFIC.^7^, ^9^, ^10^, ^11^, ^12^, ^13^, ^17^, ^18^, ^19^, ^20^, ^21^, ^22^, ^23^, ^24^, ^25^, ^26^, ^27^, ^28^

**Table 1:** ALGS and PFIC: disease characteristics and pathophysiology

	ALGS	PFIC (group of disorders)
Inheritance	Autosomal dominant	Autosomal recessive
Incidence estimate	1/30 000 to 1/50 000 live births^22^	1/50 000 to 1/100 000 live births^12^
Genetics	Mutations or deletions in *JAGGED1* or *NOTCH2*, with mutations in *JAGGED1* most common^23^, ^24^	Various genes affected that vary widely in normal function All affect bile acid transport by hepatocytes, directly or indirectly^19^, ^26^ Genetic deficiencies that produce PFIC characterized by low‐to‐normal serum GGT levels include: *ATP8B1*: the ATP8B1 protein regulates phospholipid distribution across the plasma membrane (PFIC subtype 1^a^) *ABCB11*: ABCB11, a hepatocyte bile acid transporter that exports bile salts across the canalicular membrane (PFIC subtype 2^a^) *TJP2* ^b^: encodes TJP2 or zona‐occludens 2, responsible for tight junction integrity between canalicular cells *NR1H4* ^b^: encodes FXR, the nuclear receptor that regulates expression of multiple genes related to bile transport and function, including BSEP *MYO5B* ^b^: encodes myosin 5B, important for correct localization of transporters like BSEP Above‐normal serum GGT levels are observed in *ABCB4‐*deficient PFIC, which affects MDR3, a transporter of phosphatidylcholine, a major component of bile (PFIC subtype 3^a^)
Mechanisms of disease and pathophysiology underlying cholestasis	Abnormal development of intrahepatic bile ducts and bile duct paucity^17^, ^27^	Deficient bile salt transport (due to reduced activity of ABCB11 or aberrant functioning of FXR or myosin 5B) Aberrant composition of the canalicular membrane (due to ATP8B1 deficiency), with secondary effects (eg reduced functionality of ABCB11)
Clinical presentation	ALGS is not fully penetrant (genetic confirmation necessary)^10^, ^11^ Cholestasis is common (typically presents within 3 mo of birth); usually diagnosed by age 1^9^, ^10^ Other clinical characteristics may include elevated serum bile acids, pruritus, delayed growth, distinctive facial features, renal symptoms, xanthomas and vascular anomalies^9^, ^21^	Symptom onset in ATP8B1‐ and ABCB11‐deficient patients typically occurs shortly after birth Common symptoms include discoloured stool, hepatomegaly, pruritus and/or jaundice^20^, ^25^ Additional clinical characteristics^12^, ^13^, ^20^: ATP8B1 deficiency: growth retardation and liver steatosis ABCB11 deficiency: rapid development of end‐stage liver disease
Disease progression	Estimated 10‐y survival rate among patients with ALGS born between January 1997 and May 2019:93% Native liver survival of this cohort: 70%^28^	In most cases, ATP8B1‐, ABCB11‐ and ABCB4‐deficient PFIC progress to liver failure before adulthood and are usually fatal if untreated^7^, ^12^ Mortality estimates range from 0% to 87%^c^, ^18^

Note

Higher mortality estimates may reflect disease not treated by liver transplantation.

Abbreviations: ALGS, Alagille syndrome; BSEP, bile salt export pump; FXR, farnesoid X receptor; GGT, gamma‐glutamyl transpeptidase; MDR3, multidrug resistance protein 3; PFIC, progressive familial intrahepatic cholestasis.

^a^ Historical nomenclature; current naming convention is based on genetic disruption.

^b^ Other subgroups of low‐GGT PFIC are all very rare.

^c^ Lower mortality rates may be driven by high rates of liver transplantation (range, 40%‐100% among patients with ATP8B1 or ABCB11 deficiency).

## 3 BURDEN OF CHOLESTATIC LIVER DISEASES

Previous studies have shown that patients with ALGS or PFIC have impaired quality of life, physical health and psychosocial functioning based on patient or parent proxy reports relative to healthy controls.^29^, ^30^ Intractable pruritus has been identified as the most bothersome symptom of ALGS and PFIC; its marks can be visible as scratching‐induced abrasions and scarring.^9^, ^18^, ^31^ Additional studies have highlighted that severe pruritus is associated with functional impacts such as interference with sleep or mood disturbance.^9^, ^31^, ^32^

Liver transplantation is a common treatment for patients with ALGS or PFIC. The *G*lobal *AL*agille *A*lliance (GALA) study, which was described in a congress abstract reporting on a large cohort of patients with ALGS, found that 10‐year native liver survival was no more than 70%.^28^ This is further illustrated in published data from 293 patients with ALGS and cholestasis in the multicentre, prospective *Chi*ldhood *L*iver *D*isease *Re*search *N*etwork (ChiLDReN) study, in which estimated liver transplant‐free survival was 24% at age 18.5.^33^ The experience is similar for patients with PFIC. The 10‐year native liver survival among patients with ATP8B1‐ and ABCB11‐deficient PFIC, as reported by the *NA*tural course and *P*rognosis of *P*FIC and *E*ffect of biliary *D*iversion (NAPPED) consortium, was 46%‐51%.^34^ Additional analyses from the NAPPED consortium found that the time of median native liver survival varied by underlying genotype in ABCB11‐deficient PFIC; for example, certain missense mutations that produced BSEP with residual function were associated with a median native liver survival of 20.4 years, whereas mutations that completely disrupted the BSEP protein resulted in a median native liver survival of 3.5 years.^35^ Healthcare costs in patients with PFIC and ALGS are likely considerable due to hospital visits and the need for long‐term care.^9^, ^18^

## 4 CURRENT TREATMENT LANDSCAPE AND OPPORTUNITIES FOR THERAPY

There are currently no approved drug treatments for either ALGS or PFIC. Medical treatment options are used supportively or for symptomatic relief and may include off‐label use of ursodeoxycholic acid (UDCA) to increase bile flow and reduce liver damage.^36^ Other medications are used to manage pruritus^37^ including cholestyramine, which sequesters bile acids in a resin complex for excretion^38^; rifampin, which activates the nuclear pregnane X receptor and is thought to increase the elimination of bilirubin and enhance enzymatic reactions that make bile acids more hydrophilic and less toxic^39^, ^40^, ^41^; naltrexone, an opioid antagonist used to decrease opioid‐mediated neurotransmission associated with pruritus and/or cholestasis^42^; or antihistamines. In addition, a high‐calorie diet with vitamin/mineral supplementation (eg calcium, zinc and vitamins A, D, E and K) to provide nutritional support is frequently prescribed.^43^ However, these approaches may not be entirely effective, and many patients either do not respond at all or require combination therapy.^6^, ^44^

Surgical options for treating ALGS and PFIC include surgical biliary diversion (SBD) and liver transplantation.^6^ SBD, such as partial external biliary diversion (PEBD), may be performed in patients with severe pruritus that is not effectively managed with medications.^16^ Liver transplantation is typically used for patients with end‐stage liver disease, with hepatocellular carcinoma (increased risk for its development in ABCB11 deficiency) or when other treatment options have been exhausted.^45^, ^46^, ^47^ While SBD and liver transplantation are viable treatment options for ALGS and PFIC, post‐surgery issues such as the presence of a stoma (in the case of PEBD), the need for lifelong antirejection medication (in the case of transplant) or surgical complications may be practical limitations.^15^, ^48^ Less invasive treatment options that can reduce the accumulation of bile acids in the liver and potentially relieve pruritus and cholestasis, limit the progression of liver disease and improve long‐term prognosis would be valuable.

A number of alternative, nonsurgical therapies for cholestatic liver diseases, in general, are currently under investigation. These include modalities that target the FXR‐FGF19 signalling axis (eg FXR agonists, FGF19 mimetics, obeticholic acid), cholehepatic drugs (eg norUDCA) and enterohepatic blockers (eg IBAT inhibitors).^49^ Compounds that act through FXR or FGF19 are proposed to stimulate bile acid transporter synthesis and the production of other gene products, with a cumulative effect of reducing intrahepatic bile acids level.^50^ Another promising compound is norUDCA, a derivative of UDCA that protects cholangiocytes from bile acid injury.^50^ These compounds have typically been evaluated in trials of cholestatic liver diseases in adults, and the benefits and risks to paediatric patients with ALGS and PFIC are unknown.

Potential therapies with specific applications for ALGS and PFIC include *JAGGED1* small interfering RNAs and induced pluripotent stem cells (reviewed in Feldman and Sokol,^50^ Morell and Strazzabosco,^51^ and Hansel et al^52^). In addition, there is a growing interest in IBAT inhibitors (currently in late‐stage clinical development for cholestatic liver diseases, including ALGS and PFIC) due to their specificity for IBAT in the intestine and their limited side‐effect profile outside the gastrointestinal system. IBAT inhibitors are the focus of the rest of this review.

## 5 INTERRUPTION OF THE ENTEROHEPATIC CIRCULATION AS A TREATMENT TARGET

Given that patients with ALGS and PFIC have intrahepatic accumulations of bile acids that can damage tissues in the liver and spill over into systemic circulation, SBD procedures were developed to interrupt enterohepatic circulation and reduce the bile acid pool in these patients.^8^, ^15^ SBD is often associated with reductions in serum bile acids and pruritus as well as improvements in sleep disturbance, quality of life, fibrosis and growth.^53^, ^54^, ^55^ In the case of PFIC, most of the currently available data on SBD are based on ATP8B1‐ and ABCB11‐deficient patients.^20^, ^48^, ^54^, ^56^, ^57^, ^58^

Findings from the NAPPED consortium showed that patients with ABCB11 deficiency who underwent SBD (n = 61) typically had reduced pruritus and serum bile acids relative to pre‐surgery (pruritus was present in 97% of patients prior to surgery and in 46% after SBD; mean serum bile acids decreased from 363 μmol/L initially to 48 μmol/L after SBD).^35^ Furthermore, a significant association was identified between lower post‐SBD serum bile acids and long‐term native liver survival: patients whose serum bile acids were <102 μmol/L after SBD survived up to 15 years with their native liver intact vs patients whose serum bile acids were ≥102 μmol/L, for whom less than half had this outcome.^35^ Similarly, in a systematic review and meta‐analysis evaluating studies with pre‐ and post‐PEBD liver biochemistry values, patients with PFIC with reduced serum bile acids post‐PEBD were more likely to have favourable clinical responses (ie, improved pruritus, decreased need for liver transplant).^59^ Thus, the reduction in bile acids and improvement in clinical outcomes observed with SBD provide a strong rationale that disrupting enterohepatic circulation holds promise for treating patients with cholestatic liver disease. Data from the NAPPED consortium also indicated that patient genotype, at least in the case of ABCB11‐deficient PFIC for which data are available, may influence long‐term outcomes following SBD; these data hint at a possibility for personalized medicine approaches in the future.^35^

Inhibition of IBAT represents a pharmacologic approach for achieving the same ends as SBD: that is, interruption of the enterohepatic circulation of bile acids. IBAT is an integral brush border membrane glycoprotein that co‐transports sodium and bile acids and is a major regulator of the bile acid pool size in animals and humans.^60^ IBAT inhibition prevents the intestinal reabsorption of bile acids to reduce bile acids in the liver and would be a nonsurgical alternative to SBD. Genetic ablation of IBAT in mice demonstrated that loss of IBAT function and the resulting redirection of bile acids to the colon cannot fully compensate for the increase in bile acid synthesis^61^; based on this premise, selective IBAT inhibition is thought to produce a net reduction in the hepatic exposure to bile acids (Figure 1B).^3^

One piece of evidence that IBAT inhibition could provide benefits similar to SBD is provided by a case report of a patient with ABCB11 deficiency.^62^ This patient was treated with the IBAT inhibitor odevixibat in a phase 2 clinical trial^63^ and experienced improvements in serum bile acids, pruritus and sleep. When the trial ended, the patient's symptoms returned. The patient subsequently underwent PEBD, which resulted in reductions in pruritus and serum bile acids and improvements in sleep similar to those achieved with prior IBAT inhibitor treatment, suggesting that in this patient, IBAT inhibition was as effective as PEBD for treating cholestasis.^62^

## 6 IBAT INHIBITORS IN DEVELOPMENT

Key preclinical and clinical data for 5 IBAT inhibitors in development are summarized in Table 2.^63^, ^64^, ^65^, ^66^, ^67^, ^68^, ^69^, ^70^, ^71^, ^72^, ^73^, ^74^, ^75^, ^76^, ^77^, ^78^, ^79^, ^80^, ^81^ All are selective, reversible small molecule inhibitors of IBAT, administered orally once or twice daily. Overall, study data supported the anticipated effects of IBAT inhibition, that is, decreased hepatic and circulating bile acid levels accompanied by increased fecal bile acid excretion.^82^, ^83^, ^84^, ^85^, ^86^

**Table 2:** IBAT inhibitors currently in development

IBAT inhibitor	Target indication(s)	Key preclinical findings		Key clinical findings	Current status
*Paediatric Cholestatic Liver Diseases*					
Maralixibat (LUM001; SHP625; lopixibat chloride)	ALGS; PFIC; BA (planned)	A close analogue, SC‐435, reduced bile acids and cholestatic liver injury and improved expression of proinflammatory and fibrotic markers in *Mdr2^−/−^* mice^81^ Reduced serum bile acids and liver tissue damage in rats with partial bile duct ligation (cholestasis model)^64^		The 2 highest doses did not reduce pruritus vs placebo (prespecified primary analysis), although improved pruritus was observed with the 2 lowest doses in a phase 2 trial (ITCH) for paediatric patients with ALGS (study duration, 17 wk)^65^ Serum bile acids were reduced and pruritus was improved in an interim analysis of an open‐label phase 2 study (ICONIC) in paediatric patients with ALGS (study duration, 100 wk); the most frequently reported AEs were diarrhoea, abdominal pain, vomiting and URTI^66^ Reduced serum bile acids and pruritus at week 48 in an open‐label phase 2 study (INDIGO) in children aged 1‐13 y with PFIC; treatment response up to week 72 was associated with improved growth^67^, ^68^	Orphan drug designation by FDA and EMA for ALGS, PFIC, PBC and PSC FDA breakthrough therapy designation for PFIC‐2 and ALGS Three phase 2 studies (IMAGINE [NCT02047318], IMAGINE‐II [NCT02117713], ICONIC [NCT02160782]) in ALGS are underway Phase 3 PFIC study (MARCH‐PFIC [NCT03905330]) planned
Odevixibat (A4250)	PFIC; ALGS; BA	Negative cytotoxicity; no effect on CNS, renal, GI tract, respiratory or CV parameters; well tolerated with primarily GI findings; good safety margins for projected clinical doses (data on file, Albireo Pharma, Inc) Reduced bile acids and cholestatic liver injury and improved expression of proinflammatory and fibrotic markers in *Mdr2^−/−^* mice^69^		Improved bile acids, pruritus and sleep in a phase 2, dose‐escalation, open‐label study (study duration, 8‐10 wk) that enrolled patients aged 1‐18 y with pruritus and PFIC, ALGS, BA or other causes of intrahepatic cholestasis; the most common AEs were ear infection and pyrexia, which were deemed unrelated to treatment^63^, ^70^, ^71^	Orphan drug designation by FDA and EMA for ALGS, PFIC, BA and PBC FDA fast track designation for PFIC in 2018 Phase 3 study in PFIC (PEDFIC‐1 [NCT03566238]) and an extension (PEDFIC‐2 [NCT03659916]) are underway Phase 3 study in BA (BOLD [NCT04336722]) initiated in 2020^98^
*GI, Metabolic and Other Hepatic Conditions*					
Elobixibat (A3309)	Chronic constipation; NASH	Improved constipation symptoms in dogs^72^	Demonstrated efficacy and safety for chronic idiopathic constipation in phase 2 and 3 studies in the US and Japan for up to 52 wk^73^, ^74^ Produced favourable metabolic effects vs placebo (eg decreased LDL cholesterol, increased GLP‐1) in patients with dyslipidaemia or chronic constipation (study durations, 6 and 2 wk respectively)^75^, ^92^		Received approval in Japan in 2018 for treatment of constipation A phase 2 trial for NAFLD or NASH is underway (NCT04006145)
Linerixibat (GSK2330672)	Type 2 diabetes; cholestasis; PBC	Lowered glucose levels in a diabetic rat model^76^	Reduced serum bile acids and pruritus relative to placebo in a phase 2 crossover trial of PBC in adults (study duration, 8‐14 wk); well tolerated, with diarrhoea as the most common AE^77^ Reduced glucose and lipid levels vs placebo in adults with type 2 diabetes in 2 studies (study durations, 6‐8 and 5 wk respectively); high incidence of GI‐related AEs of mild or moderate severity^78^		A phase 2 study for PBC is currently underway (NCT02966834)
Volixibat (SHP626)	NASH; ICP; PSC	Lowered cholesterol and insulin levels, reduced hepatocyte hypertrophy and increased total bile acids in faeces in a NASH mouse model^79^	Phase 2 trial in NASH patients terminated by sponsor in 2018 after no difference found vs placebo on MRI proton density fat fraction, serum ALT levels or liver histology at 24 wk^80^		FDA fast track designation for NASH in 2016 Studies for ICP and PSC are planned for 2020 (trials not yet registered)

Abbreviations: AE, adverse event; ALGS, Alagille syndrome; ALT, alanine aminotransferase; BA, biliary atresia; CNS, central nervous system; CV, cardiovascular; EMA, European Medicines Agency; FDA, United States Food and Drug Administration; GI, gastrointestinal; GLP‐1, glucose‐dependent insulinotropic peptide; IBAT, ileal bile acid transporter; ICP, intrahepatic cholestasis of pregnancy; LDL, low density lipoprotein; MRI, magnetic resonance imaging; NAFLD, nonalcoholic fatty liver disease; NASH, nonalcoholic steatohepatitis; PBC, primary biliary cholangitis; PFIC, progressive familial intrahepatic cholestasis; PSC, primary sclerosing cholangitis; URTI, upper respiratory tract infection.

IBAT inhibitors are currently in development for a range of target indications across both paediatric (eg PFIC, ALGS and others) and adult (eg PBC, PSC, others) populations. In cholestatic liver diseases, preventing the return of bile acids to the liver via IBAT inhibition may relieve the inflammatory and fibrotic pressures driving tissue damage such that cholestasis and liver function may improve.^3^, ^69^ Because IBAT inhibition results in more bile acids redirected to the colon (which stimulates colonic motility), IBAT inhibitors are also being investigated to treat constipation.^87^ Finally, because bile salts can act as signalling molecules via their interactions with nuclear receptors and downstream targets including genes involved in lipid and glucose metabolism, they may also be potentially useful in the treatment of metabolic disorders such as type 2 diabetes mellitus or nonalcoholic steatohepatitis (NASH).^88^

Two IBAT inhibitors, maralixibat and odevixibat, have been evaluated in phase 2 and phase 3 clinical trials of paediatric patients with ALGS and PFIC; however, much of the available clinical data are from results thus far only presented at scientific congresses, with 1 exception for which data from a peer‐reviewed publication are available.^65^ Maralixibat has been evaluated for ALGS in 2 phase 2 trials (ITCH^65^ and IMAGO [some study results were included with ITCH study findings]) and in three additional long‐term phase 2 trials that are ongoing (ICONIC^66^, ^89^ and IMAGINE‐I^90^ and –II, extensions of the IMAGO trial). In the ITCH trial, the group of patients with ALGS treated with the two highest doses of maralixibat did not show a difference from placebo on a measure of pruritus (assessed via the observer‐rated Itch Reported Outcome scale).^65^, ^91^ However, among all patients treated with maralixibat (3 dose groups combined), a greater proportion achieved a 1‐point pruritus score reduction than those who received placebo (68% vs 25%). The change from baseline in total serum bile acids for any maralixibat group was similar to the change observed with placebo. Gastrointestinal side effects were reported by approximately half of all patients treated with maralixibat, but none were severe. Additionally, maralixibat is being evaluated for PFIC in 2 long‐term studies: the phase 2 INDIGO^67^ study and the phase 3 MARCH‐PFIC trial.

Odevixibat was evaluated as treatment for paediatric cholestatic liver diseases, including ALGS and PFIC, in a phase 2 study.^63^ Key findings from this study include reductions in serum bile acids from baseline (with some patients experiencing up to a 98% reduction) and improvements in patient‐recorded pruritic and sleep disturbance symptoms (pruritus was assessed using 3 scales).^63^ Overall, 7/24 patients reported any gastrointestinal adverse event, and all but 1 were mild or moderate in severity. A phase 3 study and its long‐term extension study evaluating odevixibat in patients with PFIC are underway (PEDFIC‐1 and PEDFIC‐2, respectively; PEDFIC‐2 also includes a cohort of patients with other types of PFIC).

IBAT inhibitors are also in clinical development for other cholestatic liver diseases and indications, for which phase 2 and 3 data are summarized below. For the studies described below, some data were available from published abstracts only, but the majority of data were available in peer‐reviewed publications.^73^, ^74^, ^75^, ^77^, ^78^, ^92^, ^93^, ^94^, ^95^

Maralixibat has been evaluated in phase 2 trials for cholestatic liver disease in adults (PBC,^94^ PSC^96^), and a trial investigating maralixibat for biliary atresia is planned for 2020.^97^ The phase 2 study of odevixibat described above also evaluated paediatric patients with other types of cholestatic liver disease, including those with diagnoses of biliary atresia, multidrug resistance protein 3‐deficient PFIC and other causes of intrahepatic cholestasis.^63^, ^70^, ^71^ A trial investigating odevixibat for biliary atresia (BOLD; NCT04336722) started in 2020.^98^ Furthermore, odevixibat was evaluated as treatment for adults with PBC.^95^ Elobixibat is approved in Japan for the treatment of chronic constipation (supported by clinical data from a number of phase 2^73^, ^92^ and phase 3^74^ studies), and is currently being explored in a phase 2 study of patients with either nonalcoholic fatty liver disease or NASH (NCT04006145); phase 2 studies indicated metabolic effects of elobixibat.^75^ The IBAT inhibitor linerixibat demonstrated efficacy in reducing pruritus severity in adults with PBC.^77^ In another phase 2 study, pruritus in patients with PBC was associated with elevated serum bile acids and autotaxin levels, and treatment with linerixibat reduced serum bile acids.^93^ Linerixibat is currently being investigated in a phase 2 dose‐response trial of adults with PBC and pruritus (NCT02966834), and previously was evaluated in a phase 2 trial for type 2 diabetes.^78^ The IBAT inhibitor volixibat did not meet the primary efficacy endpoint in a phase 2 trial for the treatment of NASH.^80^ Finally, 2 trials to investigate volixibat in PSC and intrahepatic cholestasis of pregnancy are planned to initiate in 2020. Because bile‐modulating therapies including IBAT inhibitors are being explored for the treatment of PSC in adults,^99^, ^100^ IBAT inhibition may also be a potential therapeutic option in children with PSC; however, clinical studies are needed to determine efficacy and safety in this population.

## 7 CONCLUSIONS

ALGS and PFIC are rare, inherited childhood disorders that manifest with cholestasis and pruritus as well as progressive, life‐threatening liver disease. Limited treatments are available, and there are currently no approved pharmacologic therapies. Preclinical and clinical data support IBAT inhibitors as noninvasive options to interrupt the enterohepatic circulation to treat cholestatic liver diseases and other disorders. These orally administered, selective and reversible compounds decrease enteric bile acid reuptake with minimal systemic exposure. They may play an important role in reducing the symptoms of ALGS and PFIC by pharmacologically interrupting the enterohepatic circulation of bile acids, thus reducing bile acid accumulation in the liver and reducing the potential for hepatobiliary injury.

## CONFLICT OF INTEREST

Binita Kamath is a consultant for Albireo, Mirum and DCI. Philip Stein is an employee of Albireo Pharma, Inc. Roderick Houwen is and/or was a consultant for the Dutch Medicine Authority and GMPOrphan, Univar, Albireo and Alexion. Henkjan Verkade is a consultant for Albireo, Ausnutria, Intercept, Mirum, Vivet, FrieslandCampina Dairy Foods, GMP‐Orphan and Shire.

## ETHICS APPROVAL AND PATIENT CONSENT

Not applicable.
